# Quantum dot assisted tracking of the intracellular protein Cyclin E in *Xenopus laevis* embryos

**DOI:** 10.1186/s12951-015-0092-6

**Published:** 2015-04-29

**Authors:** Yekaterina I Brandt, Therese Mitchell, Gennady A Smolyakov, Marek Osiński, Rebecca S Hartley

**Affiliations:** Department of Cell Biology and Physiology, University of New Mexico Health Sciences Center, Albuquerque, New Mexico 87131-0001 USA; Center for High Technology Materials, University of New Mexico, 1313 Goddard SE, Albuquerque, New Mexico 87106-4343 USA

**Keywords:** Quantum dots, Intracellular protein tracking, Cyclin E, *Xenopus laevis*

## Abstract

**Background:**

Luminescent semiconductor nanocrystals, also known as quantum dots (QD), possess highly desirable optical properties that account for development of a variety of exciting biomedical techniques. These properties include long-term stability, brightness, narrow emission spectra, size tunable properties and resistance to photobleaching. QD have many promising applications in biology and the list is constantly growing. These applications include DNA or protein tagging for *in vitro* assays, deep-tissue imaging, fluorescence resonance energy transfer (FRET), and studying dynamics of cell surface receptors, among others. Here we explored the potential of QD-mediated labeling for the purpose of tracking an intracellular protein inside live cells.

**Results:**

We manufactured dihydrolipoic acid (DHLA)-capped CdSe-ZnS core-shell QD, not available commercially, and coupled them to the cell cycle regulatory protein Cyclin E. We then utilized the QD fluorescence capabilities for visualization of Cyclin E trafficking within cells of *Xenopus laevis* embryos in real time.

**Conclusions:**

These studies provide “proof-of-concept” for this approach by tracking QD-tagged Cyclin E within cells of developing embryos, before and during an important developmental period, the midblastula transition. Importantly, we show that the attachment of QD to Cyclin E did not disrupt its proper intracellular distribution prior to and during the midblastula transition. The fate of the QD after cyclin E degradation following the midblastula transition remains unknown.

**Electronic supplementary material:**

The online version of this article (doi:10.1186/s12951-015-0092-6) contains supplementary material, which is available to authorized users.

## Background

Luminescent quantum dots (QD) are semiconductor nanocrystals with unique spectroscopic properties. QD have generated much interest in the past two decades, with current and projected applications including use as FRET donors, fluorescent labels for cellular labeling, intracellular sensors, deep-tissue and tumor imaging agents, sensitizers for photodynamic therapy, and vectors for studying nanoparticle-mediated drug delivery [1]. The advantages of QD over organic dyes and genetically engineered fluorescent proteins for tagging biomolecules are their broad excitation and narrow emission spectra, brightness, resistance to photobleaching, and the fact that they can be synthesized from a single material to emit a variety of wavelengths [2]. QD have size tunable fluorescent properties, meaning that changes in QD size result in different colors of emitted light. Larger QD emit redder (lower energy) light while smaller QD emit bluer (higher energy) light. Multiple molecules can be labeled with QD of various colors and simultaneously imaged after excitation with a single UV source, which prevents overheating of cells, a quality desirable for both *in vitro* and *in vivo* applications.

The colloidal nanocrystals most often used in fundamental or applied studies are spherical, with cores varying between 15 and 120 Å in diameter. CdSe nanocrystals are prepared by reacting organometallic precursors at high temperatures in a coordinating solvent mixture. This results in capping of the inorganic core with an organic layer of trioctylphosphine/trioctylphosphineoxide mixture (TOP/TOPO) [3]. Overcoating of the CdSe core with several layers (3–5) of wider band gap semiconducting material, such as ZnS or CdS permits passivation of the core surface and produces highly luminescent CdSe-ZnS or CdSe-CdS core-shell QD [4]. In addition, the shell protects the QD from oxidation and prevents oozing of heavy metal core components into the environment, which is very important for *in vivo* applications [5]. For biological studies, QD also need to be soluble in water. Several methods have been used to render QD water soluble, such as exchange of native TOP/TOPO organic surface ligand for a water soluble surface ligand (cap exchange) [6], encapsulation within amphiphillic molecule-copolymer micelles [7], and coating with silica [8-11].

Despite the obvious optical advantages of QD-assisted visualization, most fluorescent QD labeling is being utilized either outside of cell boundaries or directly on the surface of the plasma membrane. Targeted labeling of biological molecules with QD is utilized for a variety *in vitro* applications, including using antibody-coupled QD for immunoassays, immunochromatography assays [12-16], and as biosensors that are based on QD as FRET donors [17,18]. The antibody mediated specificity approach of tagging proteins on the cell surface i.e., receptors or receptor ligands, allows for either studying their dynamics via single-particle tracking [19-21] or targeting tumor cells for detection and destruction [22-24]. QD have also been used for cell-tracking in *Xenopus laevis* embryos, either unconjugated [7], or conjugated to a nuclear localization signal [25] in order to track morphogenetic movement of cells during development.

As protein function is directly linked to its localization within the cell, accurate assessment of protein spatio-temporal dynamics is crucial. Moreover, protein localization to different compartments within a cell provides an important regulatory role. We set out to use QD to track the movement of Cyclin E, a cell cycle regulatory protein whose expression is restricted to G1/S phase of the mammalian cell cycle, where it mediates this cell cycle transition. Cyclin E has been shown to shuttle between the nucleus and cytoplasm in mammalian cells [26]. This shuttling makes sense as two of the best defined functions of Cyclin E, initiation of DNA replication and centrosome duplication, require its presence in the nucleus and cytoplasm, respectively. Cyclin E shuttling and its degradation after the G1/S transition is regulated by phosphorylation and other post-translational modification. Malfunction of mechanisms controlling Cyclin E leads to its elevation in many types of human malignancy, where it is often present throughout the cell cycle [27]. Experimental evidence indicates that Cyclin E elevation is a cause rather than an effect of tumorigenesis [28,29].

In this study, we synthesized DHLA-capped CdSe-ZnS QD, as they are not available commercially, and use them to label polyhistidine (His_6_)-tagged recombinant Cyclin E. We demonstrate feasibility of QD-assisted tracking inside cells by visualizing the intracellular localization of QD-labeled Cyclin E in live embryos in real time during early cell division cycles.

## Results

**Figure 1:**
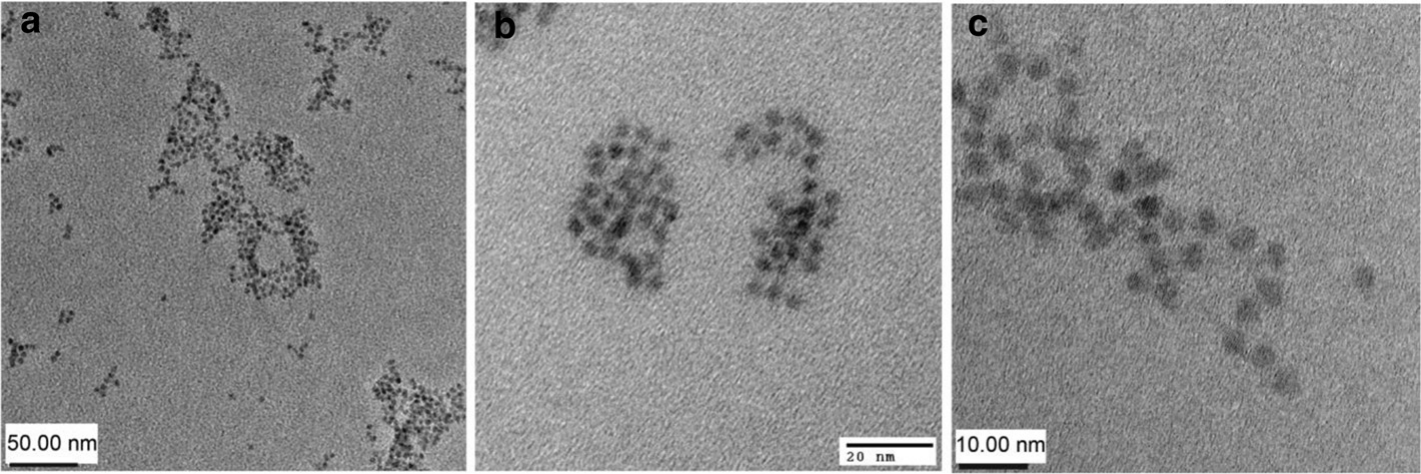
Transmission electron microphotographs of the synthesized CdSe-ZnS QD. **(a)** Scale bar is 50 nm. **(b)** Scale bar is 20 nm. **(c)** Scale bar is 10 nm.

CdSe-ZnS core-shell quantum dots with the photoluminescence emission peak of 564 nm were manufactured according to the procedure of *Clapp et al.* [4]. To make the QD soluble in water, we utilized the cap exchange strategy, exchanging TOP/TOPO with dihydrolipoic acid (DHLA). DHLA is a bifunctional ligand, containing a bidentate thiol moiety on one end, allowing its stable attachment to the inorganic QD surface, and an opposing hydrophilic end group, which permits its aqueous dispersion [4]. The DHLA-capped QD maintain their high photoluminescence and quantum yield. After synthesis and cap exchange, the morphology of DHLA coated CdSe-ZnS (QD_564_) was assessed by transmission electron microscopy (TEM). As seen in Figure 1, the QD were homogeneous, of high crystallinity (Figure 1c), consistent size (4.1 ± 0.88 nm in diameter) and shape (spherical) and did not form aggregates, as shown in Additional file 1.

**Figure 2:**
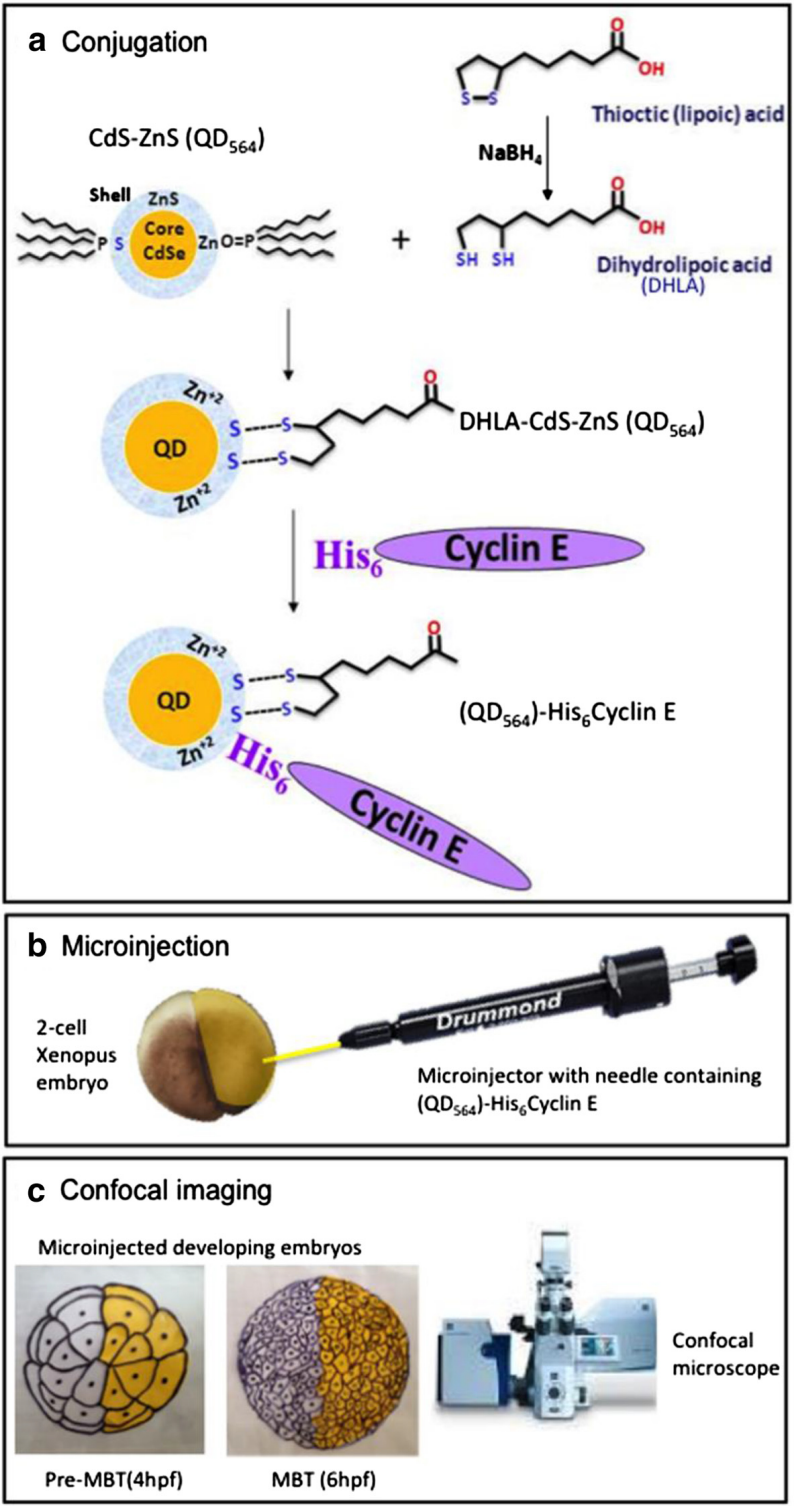
Schematic showing experimental design. **(a)** DHLA capping of CdSe-ZnS (QD_564_) and subsequent conjugation of (His_6_)-Cyclin E (modified from [5]). **(b)** Microinjection of (QD_564_)-His_6_Cyclin E into 2-cell Xenopus embryos and **(c)** confocal imaging of microinjected pre-MBT and MBT Xenopus embryos. In **(a)** the DHLA molecule contains a bidentate thiol moiety on one end, allowing its stable attachment to the inorganic QD surface. Coupling of (His_6_)-Cyclin E to QD is achieved via strong metal affinity between the histidine tag of the protein and Zn^+2^ atoms on the QD surface. The schematic is not to scale.

Next, we coupled the (His_6_)-tagged cyclin E to QD (Figure 2) and confirmed the attachment by measuring photoluminescence (PL) and quantum yield (QY). The observed 30% increase in PL intensity and 0.4% increase in QY (from 2.2% to 2.6%) of conjugated versus unconjugated QD confirmed successful bonding of protein moieties to the surfaces of the QD (Additional file 2).

(QD_564_)-His_6_Cyclin E complexes were microinjected into 1 cell of a 2-cell *Xenopus laevis* embryo between 1.5-2 hours post fertilization (hpf) and visualized in live, developing embryos by confocal microscopy. Embryos were viewed between 4 to 7 hpf (stages 6^1/2^-8), during which time embryos undergo cell divisions 5–12. This developmental period encompasses the midblastula transition (MBT), an important transition that begins around 6 hpf (stage 8) when global transcription initiates, cell motility begins, and the rapid embryonic cell cycle is remodeled to the adult cell cycle. Remodeling adds gap (G1 and G2) phases to a cell cycle that consists of rapid oscillations between DNA synthesis (S phase) and mitosis (M phase) [30]. Cyclin E is known to be nuclear at the MBT [31] but during the time we were performing these experiments, its localization before the MBT had not been determined. Therefore, we assessed Cyclin E localization before and during the MBT.

**Figure 3:**
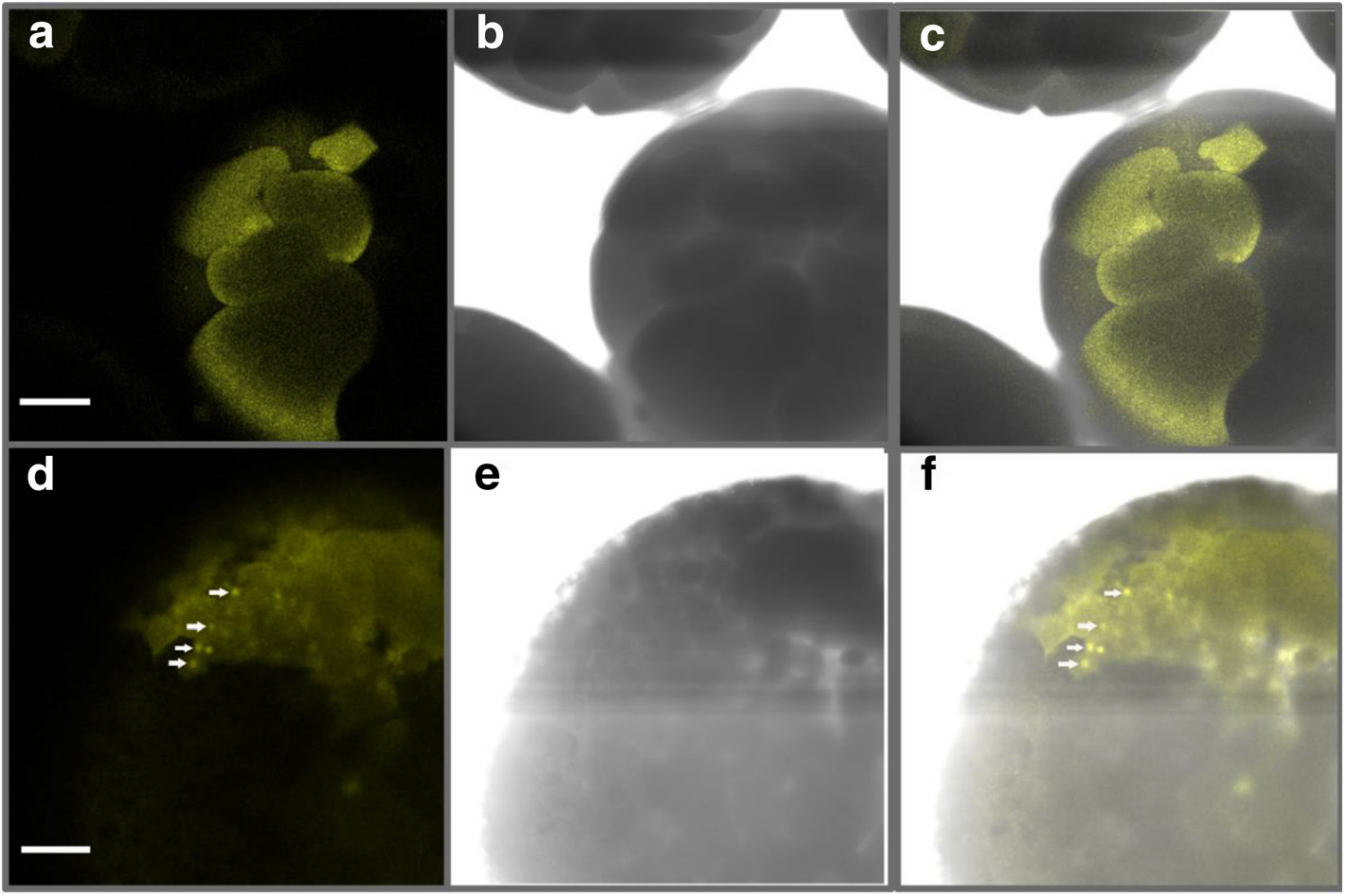
Localization of (QD_564_)-His_6_Cyclin E in live pre-MBT (4 hpf, 64-cell embryo, **a-c**) and MBT (6 hpf, 2048-cell embryo, **d-f**) *Xenopus laevis* embryos. One cell of embryos at the 2-cell stage was microinjected with (QD_564_)-His_6_Cyclin E and visualized using confocal microscopy. **(a, d)** fluorescence channel; **(b, e)** light channel; **(c, f)** merged fluorescence and light channels. Nuclei are marked with white arrowheads in panels b, f. Embryos were viewed with a 10X objective on a Zeiss LSM 510 confocal microscope equipped with a META detector, and analyzed using LSM510 Image Acquisition software. Scale bars are 100 μM. At least 20 embryos were injected and viewed in at least 3 separate experiments.

Images of embryos taken at 4 hpf, about 2 hrs post-injection (Figure 3a and c) show that localization of (QD_564_)-His_6_Cyclin E was cytoplasmic prior to the MBT, as evident from the diffuse staining seen within individual blastomeres (large cells). At the MBT (6 hpf), (QD_564_)-His_6_Cyclin E began to accumulate in the nuclei of the cells (Figure 3d and f) (white arrows). The daughter cells that originated from the uninjected cell of the 2-cell embryo lack luminescence and serve as a negative control for QD detection, as only cells derived from the injected cell should contain the QD. The cells derived from the uninjected blastomere can be clearly seen as they lack luminescence in Figure 3c and f, which show merged images of the UV and light channels. Gap junctions and cytoplasmic bridges couple cells of the early embryo during cleavage [32,33]. Gap junctions are too small for QD to cross (1–2 nm). Lack of QD luminescence in the sister cell at 2-cell or 4-cell stages (not shown), as well as in a very specific subset of cells at later stages (Figure 3), show that QD are not diffusing via gap junctions or cytoplasmic bridges (QD are injected after the first cleavage division is complete). It should also be noted that individual cells of the embryo become progressively smaller as embryos continue to divide without growing during this developmental timeframe (see Figure 2c for a graphical illustration).

**Figure 4:**
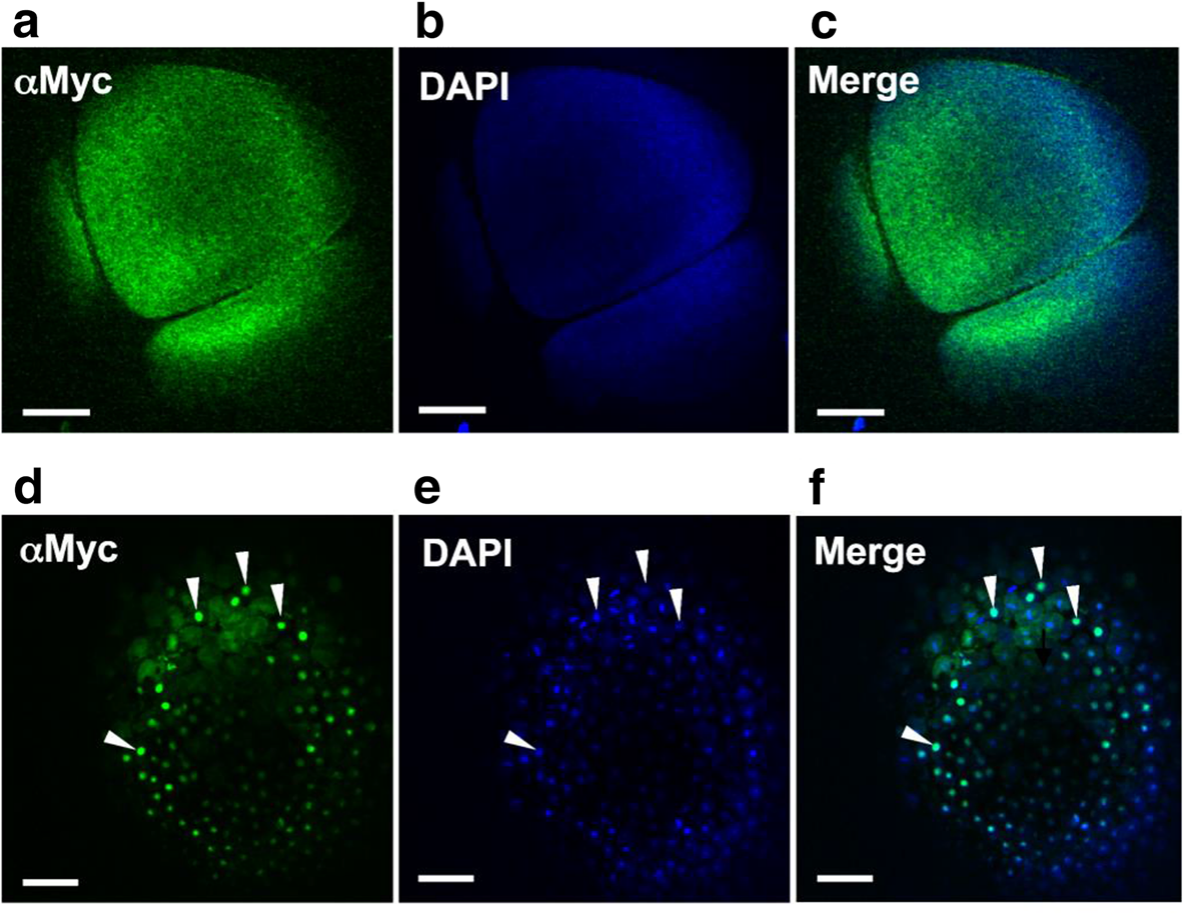
Localization of exogenous Cyclin E in pre-MBT and MBT *Xenopus laevis* embryos. One cell of 2-cell embryo was microinjected with *in vitro* transcribed Myc_6−_GFP-Cyclin E RNA, collected at indicated time points, and the translated protein detected in fixed and stained embryos. For immunofluorescence analysis of Cyclin E localization, embryos were collected at 4 hpf, pre-MBT **(a-c)** or at 6 hpf, MBT **(d-f). (a, d)** Embryos were fixed and stained with an antibody against the Myc_6_ tag (αMyc) followed by an Alexa488 conjugated secondary antibody. **(b, e)** Embryos were counterstained with DAPI to visualize the nuclei. **(c, f)**. Merged image of the Alexa488 and DAPI. White arrowheads in d-f indicate nuclei. Embryos were viewed with a 10X objective on a Zeiss LSM 510 confocal microscope equipped with a META detector, and analyzed using LSM510 Image Acquisition software. Scale bars are 100 μM. At least 20 embryos were injected in at least 3 separate experiments, with at least 5 embryos fixed per timepoint for analysis.

Our tracking results of Cyclin E labeled with QD are in agreement with a previous study that reported nuclear localization of Cyclin E at the MBT [31]. They are also consistent with our published work showing the localization pattern of endogenous and exogenous Cyclin E [34]. Similarly to these published results, Figure 4 shows immunofluorescence staining of Myc_6_-GFP-Cyclin E in embryos, produced from *in vitro* transcribed mRNA also injected at the 2-cell stage. The embryos were probed with an antibody to the Myc_6_-tag followed by an Alexa Fluor488 conjugated secondary antibody. Exogenous Cyclin E was diffusely distributed in the cytoplasm pre-MBT (Figure 4a) and accumulated in the nuclei at the MBT in fixed embryos (Figure 4d). Figure 4b shows the diffuse distribution of DAPI in the pre-MBT embryos, due to its inability to diffuse though the lipid-rich cytoplasm even in fixed embryos. Figure 4c is a merged image of the Alexa Fluor488 and DAPI channels. Figure 4e shows DAPI stained nuclei in an MBT embryo (white arrowheads), while Figure 4f shows the coincidence of DAPI and exogenous cyclin E. DAPI easily stains the nuclei in MBT embryos as the ratio of cytoplasm to nucleus is greatly reduced in the smaller cells. Cells in mitosis (identified by the condensed chromatin and mitotic figures) have diffusely distributed cytoplasmic cyclin E (Figure 4d-f), also in agreement with previous studies [30].

**Figure 5:**
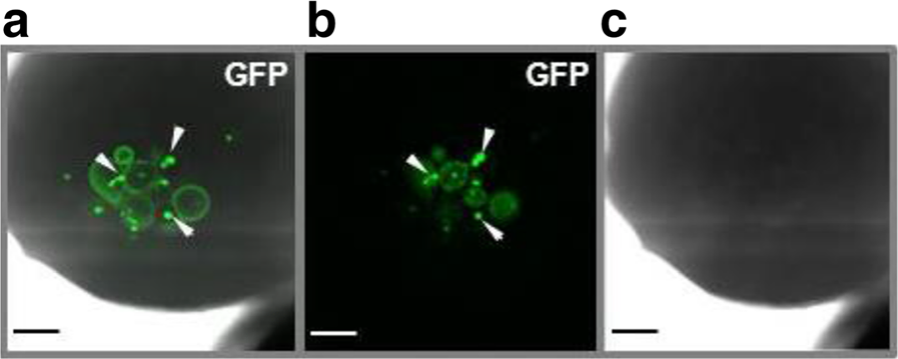
Cyclin E accumulates in the nucleus of live *Xenopus laevis* embryos at the MBT (6 hpf). One cell of the 2-cell embryo was microinjected with *in vitro* transcribed Myc_6_-GFP-Cyclin E RNA and the translated protein visualized in live embryos using confocal microscopy in real time. **(a, b)** Fluorescence channel, Z stack images #5 and #8 from the top, respectively. Nuclei are marked with white arrowheads. **(c)** Light field image. A 3D image is shown. Scale bars are 100 μM. Embryos were viewed with a 10X objective on a Zeiss LSM 510 confocal microscope equipped with a META detector, and analyzed using LSM510 Image Acquisition software. At least 20 embryos were injected in at least 3 separate experiments.

Additional GFP imaging of fluorescent Myc_6_-GFP-Cyclin E in live embryos confirmed its residence in the nucleus at the MBT in merged (Figure 5a) and fluorescence (Figure 5b) images (white arrowheads). Altogether, these results provide evidence that attachment of QD to Cyclin E leads to excellent tracking capabilities, without altering the ability of a protein to follow a proper and timely localization pattern intracellularly.

We also assessed potential cytotoxicity of the (QD_564_)-His_6_Cyclin E complexes by carefully monitoring embryo morphology and survival for up to 3 days after microinjection. In a typical experiment, 20 embryos each were microinjected with either QD solution or solvent (water). Of these 20 embryos, 10–15 survived in both the QD-injected and Solvent-injected embryos. Of these, approximately 6–10 developed into free-swimming tadpoles. This number varied depending on the quality of the eggs and sperm. Germ cell quality varies with age of the female and male frogs, the number of times the female frog has been ovulated, as well as environmental factors such as temperature. Injected embryos progressed through the blastula stage, gastrulated normally, and became free-swimming tadpoles. We were able to visualize QD-mediated fluorescence in embryos microinjected with (QD_564_)-His_6_Cyclin E complexes for up to three days after microinjection without any obvious deleterious effects. These results suggest that toxicity associated with the proposed application of QD-assisted tracking in Xenopus embryos remained low. This low toxicity differs from reports using commercial QD in Xenopus embryos, which showed variable cytotoxicity [25], likely due to variable overcoating. The low toxicity of our QD is probably due to consistent overcoating, which can be controlled when they are synthesized in lab.

## Discussion

In this paper we conducted “proof-of-principle” experiments for an exciting application for engineered nanoscale products in the biomedical field, tracking of intracellular protein inside cells. We accomplished both objectives we set out to achieve. First, we successfully synthesized functional DHLA-capped CdSe-ZnS QD specifically tailored to our application of attachment of recombinant (His_6_)-Cyclin E protein to its surface. Second, we followed our QD-tagged Cyclin E complexes inside the cells of developing Xenopus embryos in real time, which allowed us to discriminate between cytoplasmic Cyclin E localization before the MBT (4 hpf) and its accumulation in the nucleus at the MBT (6 hpf). Therefore we provide evidence for the feasibility of this approach.

For our experiments we chose ZnS overcoated CdSe quantum dots capped with DHLA. Cap exchange with DHLA not only renders QD water soluble [35], but also allows direct attachment of Histidine-tagged proteins. Alternatively, a more traditional conjugation approach involves the use of the EDC (1-ethyl-3-(3-dimethylaminopropyl) carbomide) crosslinking agent that reacts with carboxyl groups on the QD surface to the primary amine groups of the protein. The direct coupling strategy that we utilized has several unique advantages. First, it allows a unidirectional attachment of protein to the QD. Second, QD-protein complexes are less prone to aggregation in neutral and acidic buffers. Third, the attachment of His-tagged proteins to the DHLA capped quantum dots is mediated by a strong metal affinity between the His_6_-tag and Zn^2+^ atoms of the QD shell [5] (Figure 2), with a dissociation constant (K_d_) of 1 × 10 ^−13^. This interaction is stronger than most antibody binding, which has a K_d_ of 1×10^−6^-10^−9^ [36].

Unlike most mammalian cells, *Xenopus laevis* embryos provide an ideal model for a straightforward and efficient intracellular delivery of QD-protein complexes. Since QD-His_6_-protein complexes are stable at physiological pH, they can be microinjected into the 1- or 2- cell (or even later), 1–1.5 mm diameter Xenopus embryo and imaged in real time *in vivo*. One advantage of self-synthesized QD is that the synthesis, overcoating and capping can be carefully controlled, resulting in low cytotoxicity after microinjection. Another advantage is that QD are too large to pass through the numerous gap junctions that couple cells in the early embryo [32,33], allowing tracking of only the progeny of the injected cell. One disadvantage of imaging live Xenopus embryos, is that it is very difficult to stain and image the nuclei. DAPI and other dyes cannot efficiently diffuse through the lipid rich cytoplasm, necessitating fixation and staining of the embryos. Visualizing the nuclei in living embryos can be accomplished by producing transgenic frogs expressing GFP-tagged nuclear membrane proteins [37] or by coupling QD to a nuclear localization signal [25], which in our case would have interfered with monitoring cyclin E intracellular localization.

QD were detected in the embryos past the time that endogenous and exogenous cyclin E are normally degraded [34]. Based on our prior studies, we assume that the cyclin E1 conjugated to the QD was likely degraded following the MBT but that the QD remain. It is possible that cyclin E conjugated to the QD is not phosphorylated and therefore not degraded at the developmentally appropriate time. As the time period of interest to us in this study was prior to the time that cyclin E is degraded physiologically, we did not ask if cyclin E was degraded with normal kinetics. This is an intriguing question that will be pursued in future studies.

## Conclusions

Our approach provides an excellent alternative to the more commonly used traditional method of genetically encoded fluorophores, with its sometimes cumbersome and labor intensive cloning steps and also eliminates the risks of fusing the large protein moiety to the native protein (~30 kDa in case of GFP, YFP, and RFP) that can lead to protein misfolding, missorting, loss of fluorescent properties, or even changes in the host protein behavior and activity. Even though failures are seldom reported, many representative examples of altered fusion protein integrity are present in the literature [38-44]. Moreover, the QD-based approach is highly beneficial due to inherent fluorescent signal longevity of QD that allow deeper imaging during prolonged periods of time throughout embryo development with the additional capabilities of cell lineage tracing. It is widely applicable to other developmental systems such as zebrafish, Drosophila, *Caenorhabditis elegans*, and mouse blastocysts as well as to attached and suspended cells where cytoplasmic/nuclear microinjections are either desirable or permissible [45]. With the future advances in methods for cargo delivery inside eukaryotic/mammalian cells across lipid bilayers and the commercial availability of various QD products, the application of this method will be vastly expanded.

## Methods

### CdSe-ZnS quantum dot preparation

QD were prepared using a stepwise approach consisting of core CdSe nanocrystal growth, overcoating with five layers of ZnS, size selective precipitation, and surface ligand exchange and purification [36]. Growth of nanocrystals was monitored by a change in the absorption spectrum by UV–VIS spectroscopy. QD were made water soluble through exchanging the native capping shell (trioctylphosphine (TOP)/trioctylphosphine oxide(TOPO)/hexadecylamine) (Sigma Aldrich, St. Louis, MO) with freshly prepared DHLA [4] . DHLA was prepared by ring opening of the DHLA precursor, thioctic acid (Sigma Aldrich, St. Louis, MO) using NaBH_4_ (Fisher, Waltham, MA) as a reducing agent in aqueous solution (Figure 2) [4,46] followed by DHLA distillation to remove impurities. The QD morphology was assessed by transmission electron microscopy.

### Optical characterization

UV–VIS absorption spectra were obtained at room temperature using a Varian Carey 400 Spectrophotometer. Each aliquot was quenched directly in a UV cell containing toluene and the spectra were collected from 300 to 800 nm. QD photoluminescence (PL) and quantum yield (QY) were measured on a Horiba Jobin-Yvon Fluorolog-3 Spectrofluorometer with the excitation and emission monochromators both set at 1 nm. Additional file 2 shows results of this analysis. The optimal excitation wavelength of 450 nm was determined from PL excitation spectroscopy measurements. QY measurements were performed as described [47] using the Fluorolog-3 integrating sphere attachment and a liquid sample holder. QY determination involves five separate spectral measurements: three excitation scatter spectra taken for 1) an empty integrating sphere, 2) integrating sphere with the sample inside directly hit with the excitation light, 3) integrating sphere with the sample excited indirectly by the excitation light scattered by the integrating sphere; and two photoluminescence spectra taken for 4) the sample inside the integrating sphere directly hit by the excitation light, and 5) the sample inside the integrating sphere excited indirectly by the excitation light scattered by the integrating sphere.

### Transmission electron microscopy

Transmission electron microscopy (TEM) was used to verify size and quality of the cap-exchanged QD. An aliquot of the CdSe-ZnS dots was placed onto a carbon coated TEM grid (300 mesh) and allowed to dry for either 0.5 hour or overnight. The QD were imaged using either a Phillips CM 30 TEM at 300 kV accelerating voltage (Figure 1a and c) or a Hitachi H7500 equipped with an AMT XR60 camera (Figure 1b). Image J software was used to determine QD size, which was 4.1 ± 0.88 nm (average of 20 non overlapping QD with visible borders).

### Recombinant protein purification and conjugation to QD

The full-length open reading frame of Cyclin E was subcloned into Pepex vector [31], expressed in *E. coli,* grown at 37°C to an OD600 of 0.7, then induced with a final concentration of 1 mM of isopropyl-**β**-D-thiogalactopyranoside (Promega, Madison, WI) and grown to an OD600 of 1. The Pepex vector contains a DNA sequence specifying a string of six histidine residues at the amino terminus of the inserted coding region for a protein of interest. The result is expression of a recombinant protein with a 6xHis tag fused to its amino-terminus. *E. coli* cells were lysed followed by centrifugation and the supernatant loaded onto BioRad (Hercules, CA) econo-pack columns filled with Ni-NTA agarose (Qiagen, Ventura, CA) for purification (according to the manufacturer’s specifications). His_6_-Cyclin E was eluted by addition of phosphate buffered saline containing imidazole (Sigma-Aldrich, St. Louis, MO). Protein quality and quantity were assessed by SDS-PAGE and visualization on Coomassie stained gels. (His_6_)-Cyclin E attachment to DHLA capped QD was carried out by addition of an equal amount of (His_6_)-Cyclin E (in phosphate buffered saline) to QD in an aqueous solution of 10 mM sodium tetraborate buffer (pH 9.5); the mixture was incubated for 15 min at room temperature. pH was tightly controlled to 9.5 during all steps.

### QD intracellular delivery and microscopy

*X. laevis* embryos were obtained by inducing egg laying with hormones followed by in vitro fertilization using standard methods [48], except albino females were used for egg laying. Fertilized embryos were dejellied using 2% cysteine–HCl, pH 7.8, then maintained in 0.1X Marc’s Modified Ringer’s (0.1X MMR). Microinjections were performed in 4% Ficoll in 0.33X MMR [49]. Embryos were microinjected into the animal pole of one cell at the two-cell stage with 27.6 nl of (QD_564_)-His_6_Cyclin E. Embryos were staged according to Nieuwkoop and Faber [50] Twenty embryos were microinjected, placed in depression slides containing 0.1X MMR in 3% Ficoll and imaged live at the stated time post-fertilization. An empirically chosen constant exposure time was used for imaging on a Zeiss LSM510 confocal microscope equipped with a META detector and analyzed using LSM510 Image Acquisition software. The Z step size was 70.71 μm. The pixel size was 2.54 μm × 2.54 μm.

### Immunofluorescence analysis

The Cyclin E coding sequence (Genbank accession no. Z13966) in the pCS2mt-GFP vector (Addgene, Cambridge, MA) was used to express Myc_6_-GFP-tagged cyclin E (Myc_6_-GFP-Cyclin E), in Xenopus embryos for live imaging (Figure 5) or for immunofluorescence analysis (Figure 4), as previously described [34]. pCS2mt-GFP-cyclin E plasmid was linearized with Not I and transcribed with SP6 RNA polymerase according to the manufacturer’s instructions (Promega, Madison, WI). 1–2 μg of the in vitro transcribed RNA was analyzed on a formaldehyde gel to check quality. Capped RNA was injected into 1-cell of a 2-cell embryo (0.5 ng), between 1.5–2 hours post-fertilization. Five embryos were collected at the indicated time points and either imaged live or fixed in 3.7% formaldehyde/phosphate buffered saline (PBS) for 2 hours on a Nutator shaker. Embryos were then transferred into Dent’s fixative (4 parts of MeOH and 1 part DMSO), and incubated at − 20°C for at least 48 hours. Embryos were rehydrated in a graded series of methanol and washed twice for 10 min in PBS. Rehydrated embryos were hemi-sectioned and all incubations and washes were performed at 4°C on a Nutator. As GFP fluorescence could not be easily detected in the fixed embryos (not shown), hemi-sectioned embryos were next incubated in anti-Myc (1:2,000; Cell Signaling Technology, Danvers, MA) in PBS containing 0.05% Tween20 (PBT) overnight in order to detect the Myc tag. The embryos were washed four times with PBT, and incubated in Alexa Fluor 488 conjugated goat-anti-mouse (Molecular Probes, Eugene, OR) in PBT (1:200) overnight. Embryos were washed 3 times with PBT, nuclei counterstained with 4’,6’-diamidino-2-phenylindole (DAPI) in PBS (1:3,000 dilution of 1 mg/ml) for 30–60 min, washed extensively with PBS, and mounted in depression slides using Vectashield mounting media (Vector Laboratories, Burlingame, CA) and coverslipped. Empirically chosen acquisition parameters was used for imaging on a Zeiss LSM 510 confocal microscope equipped with a META detector, and analyzed using LSM510 Image Acquisition software. The Z step size was 70.71 μm. The pixel size was 2.54 μm × 2.54 μm.

## Additional files

Additional file 1: Figure S1. DHLA coated CdSe-ZnS (QD_564_) solution. (a) Photograph of 0.6 ml clear-walled PCR tubes containing solvent (water, left) or quantum dot solution (right), showing uniform quantum dot dispersal. (b) Stereomicroscopic image of droplets of solvent (water, left) or quantum dot solution (right) under bright field illumination. The dotted circles on the droplets are a reflection of the ring light on the stereomicroscope. Scale bar is 3 mm. (c) Stereomicroscopic image of droplets of solvent (water, left) or quantum dot solution (right) under UV illumination. Identical field is shown, except (b) is bright field and (c) UV illumination. Droplets were viewed using a Leica MZ FLIII fluorescence stereomicroscope equipped with a Leica GFP1 filter set (ex 425/60, em 480 long pass). Photographs were taken with a Photometrics Coolsnap ES camera. Additional file 2: Figure S2. (a, b) Photoluminescence spectra of DHLA-CdSe-ZnS (QD_564_) before (a) and after (b) conjugation to (His_6_) Cyclin E. (c, d ) Results of quantum yield measurements of DHLA-CdSe-ZnS (QD_564_) before (c) and after (d) conjugation to (His_6_)Cyclin E represented by excitation scatter spectra (upper panel) and the corresponding PL spectra (lower panel) of the samples. The excitation scatter spectra show the actual spectral content of the excitation source (Xenon lamp) scattered by the integrating sphere.

## Abbreviations

QD: Quantum dot
DHLA: Dihydrolipoic acid
FRET: Fluorescence resonance energy transfer
DNA: Deoxyribonucleic acid
TOP: Trioctyl phosphine
TOPO: Trioctylphosphineoxide
PL: Photoluminescence
QY: Quantum yield
His_6_: Six histidine
MBT: Mid blastula transition
hpf: Hours post fertilization
Myc_6_: Six myc
GFP: Green fluorescent protein
