# Supplementary material for: Quantum dot assisted tracking of the intracellular protein Cyclin E in Xenopus laevis embryos
Source: J Nanobiotechnology. 2015 Apr 29;13:31. doi: 10.1186/s12951-015-0092-6 (PMC4424550; doi:10.1186/s12951-015-0092-6)
Supplement: Additional file 1: Figure S1. — DHLA coated CdSe-ZnS (QD564) solution. (a) Photograph of 0.6 ml clear-walled PCR tubes containing solvent (water, left) or quantum dot solution (right), showing uniform quantum dot dispersal. (b) Stereomicroscopic image of droplets of solvent (water, left) or quantum dot solution (right) under bright field illumination. The dotted circles on the droplets are a reflection of the ring light on the stereomicroscope. Scale bar is 3 mm. (c) Stereomicroscopic image of droplets of solvent (water, left) or quantum dot solution (right) under UV illumination. Identical field is shown, except (b) is bright field and (c) UV illumination. Droplets were viewed using a Leica MZ FLIII fluorescence stereomicroscope equipped with a Leica GFP1 filter set (ex 425/60, em 480 long pass). Photographs were taken with a Photometrics Coolsnap ES camera. [file 12951_2015_92_MOESM1_ESM.docx]

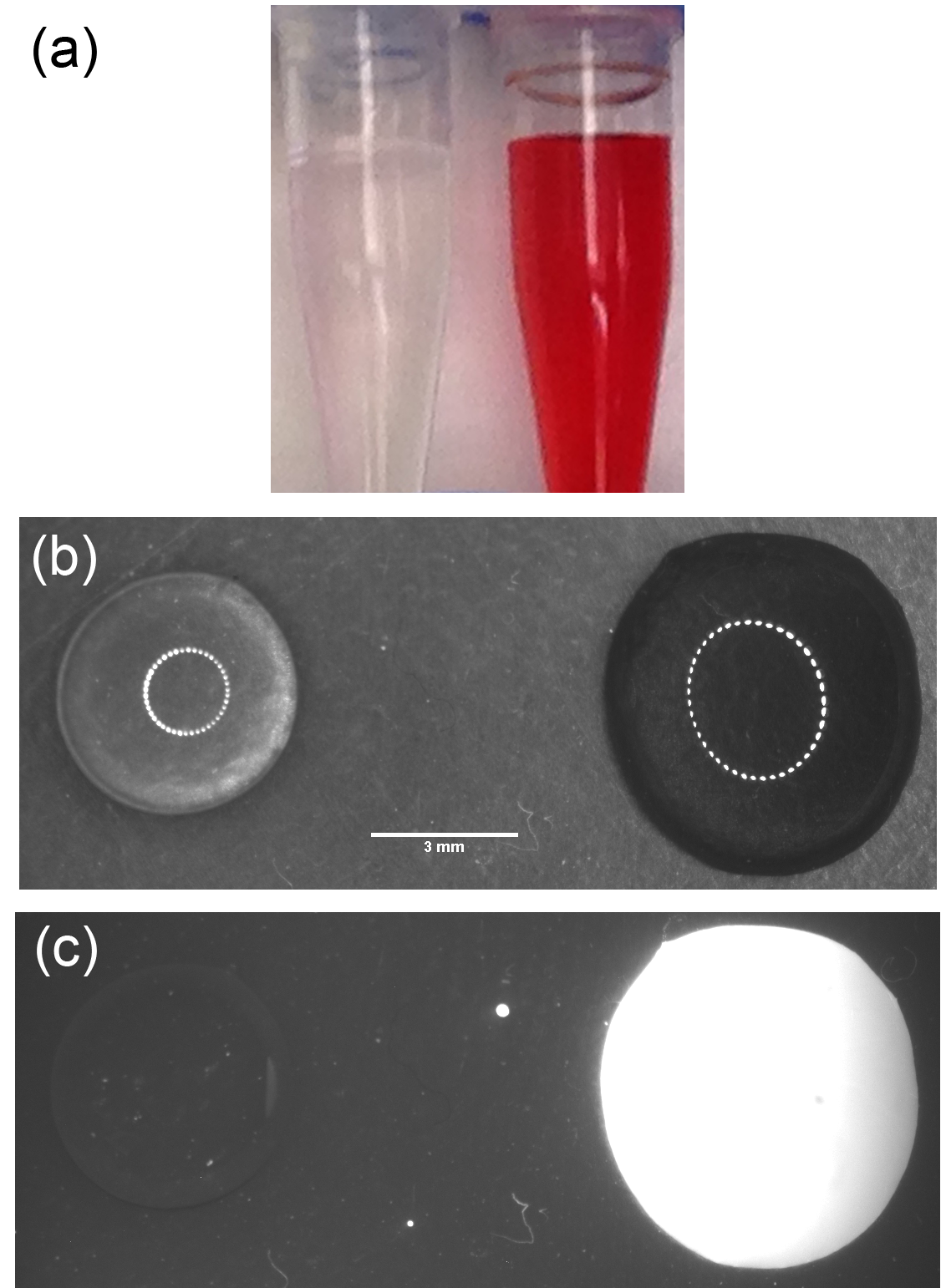


Figure S1. DHLA coated CdSe-ZnS (QD_564_) solution. (a) Photograph of 0.6 ml clear-walled PCR tubes containing solvent (water, left) or quantum dot solution (right), showing uniform quantum dot dispersal. (b) Stereomicroscopic image of droplets of solvent (water, left) or quantum dot solution (right) under bright field illumination. The dotted circles on the droplets are a reflection of the ring light on the stereomicroscope. Scale bar is 3 mm. (c) Stereomicroscopic image of droplets of solvent (water, left) or quantum dot solution (right) under UV illumination. Identical field is shown, except (a) is is bright field and (b) UV illumination. Droplets were viewed using a Leica MZ FLIII fluorescence stereomicroscope equipped with a Leica GFP1 filter set (ex 425/60, em 480 long pass). Photographs were taken with a Photometrics Coolsnap ES camera.
