# Supplementary material for: Quantum dot assisted tracking of the intracellular protein Cyclin E in Xenopus laevis embryos
Source: J Nanobiotechnology. 2015 Apr 29;13:31. doi: 10.1186/s12951-015-0092-6 (PMC4424550; doi:10.1186/s12951-015-0092-6)
Supplement: Additional file 2: Figure S2. — (a, b) Photoluminescence spectra of DHLA-CdSe-ZnS (QD564) before (a) and after (b) conjugation to (His6) Cyclin E. (c, d ) Results of quantum yield measurements of DHLA-CdSe-ZnS (QD564) before (c) and after (d) conjugation to (His6)Cyclin E represented by excitation scatter spectra (upper panel) and the corresponding PL spectra (lower panel) of the samples. The excitation scatter spectra show the actual spectral content of the excitation source (Xenon lamp) scattered by the integrating sphere. [file 12951_2015_92_MOESM2_ESM.doc]

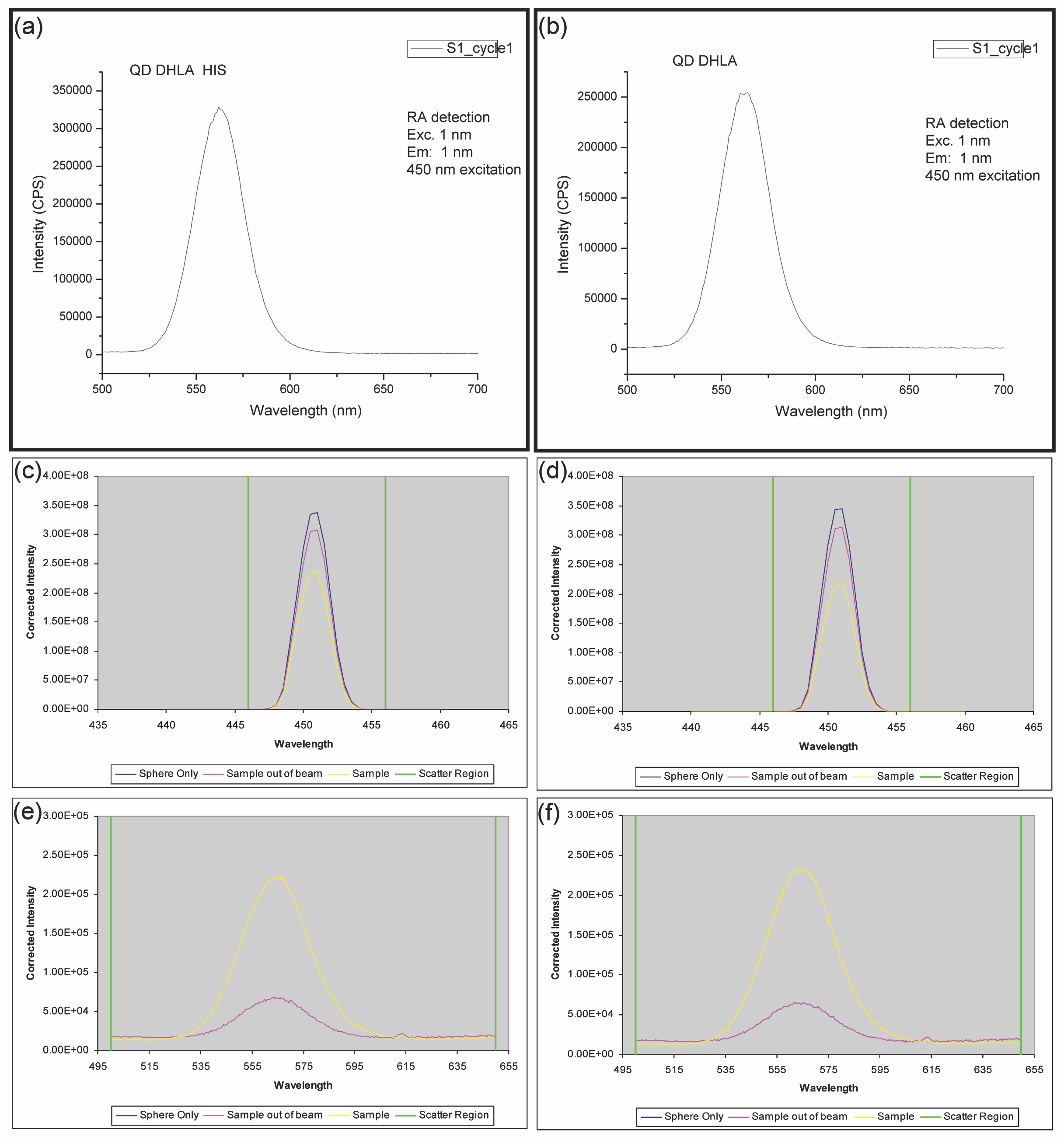


Figure S2. Excitation scatter spectra (a, b) and the corresponding photoluminescence spectra (c-f) of DHLA-CdSe-ZnS (QD_564_) before and after conjugation to (His_6_)Cyclin E. (a, c, e) conjugated (QD_564_)-His_6_Cyclin E. (b, d , f) unconjugated DHLA-CdSe-ZnS (QD_564_). Quantum yield (QY) measurements of CdSe-ZnS QDs were performed as described [43] using the Fluorolog-3 integrating sphere attachment and a liquid sample holder . QY determination involves five separate spectral measurements: three excitation scatter spectra taken for 1) an empty integrating sphere, 2) integrating sphere with the sample inside directly hit with the excitation light, 3) integrating sphere with the sample excited indirectly by the excitation light scattered by the integrating sphere; and two photoluminescence spectra taken for 4) the sample inside the integrating sphere directly hit by the excitation light, and 5) the sample inside the inegrating sphere excited indirectly by the excitation light scattered by the integrating sphere.
